# Peripheral Mononuclear Cells Metabolomics in Obstructive Sleep Apnea Syndrome (OSAS): An Exploratory Pilot Study of Immunometabolic Signatures

**DOI:** 10.3390/ijms27177658

**Published:** 2026-08-26

**Authors:** Nour Balasan, Michela Zorzi, Blendi Ura, Antonietta Robino, Paolo Dalena, Riccardo Addobbati, Mariateresa Di Stazio, Alessandro Zago, Adamo Pio d’Adamo, Domenico Leonardo Grasso, Alberto Tommasini, Egidio Barbi, Feras Kharrat

**Affiliations:** 1 Institute for Maternal and Child Health, IRCCS “Burlo Garofolo”, 34137 Trieste, Italy; nour.balasan@burlo.trieste.it (N.B.); michela.zorzi@burlo.trieste.it (M.Z.); antonietta.robino@burlo.trieste.it (A.R.); paolo.dalena@burlo.trieste.it (P.D.); riccardo.addobbati@burlo.trieste.it (R.A.); mariateresa.distazio@burlo.trieste.it (M.D.S.); alessandro.zago@burlo.trieste.it (A.Z.); adamopio.dadamo@burlo.trieste.it (A.P.d.); domenicoleonardo.grasso@burlo.trieste.it (D.L.G.); alberto.tommasini@burlo.trieste.it (A.T.); egidio.barbi@burlo.trieste.it (E.B.); feras.kharrat@burlo.trieste.it (F.K.); 2 Department of Medicine, Surgery and Health Sciences, University of Trieste, Strada di Fiume 447, 34149 Trieste, Italy

**Keywords:** obstructive sleep apnea syndrome (OSAS), PMNCs, immunometabolomics, hypoxia

## Abstract

Obstructive Sleep Apnea Syndrome (OSAS) is associated with systemic inflammation and immune dysfunction, yet the specific metabolic alterations within immune cells remain poorly understood. In this exploratory study, we characterized the metabolome of peripheral mononuclear cells (PMNCs) from patients with OSAS and healthy controls to investigate potential immunometabolic signatures associated with the disease. Initial analyses identified 21 nominally differentially abundant metabolites between OSAS patients and controls, suggesting trends such as the depletion of carnitines and alterations in fatty acid and neurotransmitter metabolism. However, following correction for multiple testing (False Discovery Rate, FDR), only putatively annotated cis,cis-muconic acid remained statistically significant. Furthermore, pathway enrichment analysis highlighted exploratory trends primarily associated with SLC-mediated transmembrane transport and energy metabolism. Our findings indicate that while OSAS PMNCs exhibit metabolic shifts suggestive of cellular stress, most of these alterations represent nominal trends that require validation in larger cohorts. The robust identification of putatively annotated cis,cis-muconic acid highlights a potential target of interest. In this exploratory pilot study, given the absence of polysomnographic and objective hypoxia parameters in most participants, observed metabolic alterations are described as associated with, rather than directly caused by, intermittent hypoxia. Overall, this study provides a hypothesis-generating framework for future research on the immunometabolic consequences of OSAS.

## 1. Introduction

Obstructive sleep apnea syndrome OSAS is the most common form of sleep disordered breathing in children. It is characterized by recurrent episodes of partial or complete upper airway obstruction during sleep, leading to intermittent hypoxia (IH) and sleep fragmentation [1]. The systemic consequences of pediatric OSAS extend well beyond the upper airway, encompassing cardiovascular morbidity, neurocognitive deficits, metabolic dysregulation, and chronic low-grade systemic inflammation. Elevated inflammatory markers, including C-reactive protein (CRP), interleukin-6 (IL-6), interleukin-8 (IL-8), and tumor necrosis factor-α (TNFα), have been consistently documented in affected children [2], alongside a characteristic Th17/Treg imbalance in peripheral blood. Th17 frequencies are markedly elevated in these children and are positively correlated with disease severity [3]. Oxidative stress is a hallmark of the condition, with decreased reduced glutathione, elevated oxidized LDL, and increased lipid peroxidation markers in the circulation of pediatric OSAS patients [4,5].

Although the metabolic consequences of OSAS have been increasingly recognized, the vast majority of metabolomic investigations to date have focused on biofluids, primarily serum, plasma and urine, in order to characterize the metabolic disturbances associated with the disease [6,7]. In an innovative study, Xu et al. identified 57 differentially expressed metabolites in the urine of 30 pediatric OSAS patients compared to controls. These metabolites included amino acids, carbohydrates, microbial metabolites, vitamins, nucleic acids, fatty acids, butanoates, and metabolites involved in bilirubin metabolism and the ornithine cycle [8]. In the adult population, several plasma and serum metabolomic studies have characterized metabolic signatures of OSAS, identifying alterations in glycerophospholipids, sphingomyelins, acylcarnitines, branched-chain amino acids, and markers of oxidative stress [9,10]. A recent study of intestinal metabolites in children aged 4–6 years with OSAS identified 254 differential metabolites, predominantly involving fatty acid, amino acid, and glucose metabolism [11]. However, none of these prior studies have examined the metabolome of the immune cells themselves, which are both sensors and effectors of the inflammatory response driven by intermittent hypoxia.

The rationale for interrogating the white blood cell metabolome is grounded in the emerging field of immunometabolomics, which recognizes that metabolic reprogramming is a fundamental feature of immune cell activation and that the metabolic fingerprint of immune cells reflects the type and duration of the immune response [12]. In OSAS, intermittent hypoxia stabilizes hypoxia-inducible factor 1-alpha (HIF-1α), a master transcription factor that reprograms cellular metabolism from oxidative phosphorylation toward glycolysis in immune cells, fundamentally altering their nutrient uptake and effector functions [13]. Ryan et al. demonstrated that intermittent hypoxia selectively activates the NF-κB inflammatory pathway over the HIF-1α adaptive pathway in circulating leukocytes, thereby promoting pro-inflammatory cytokine production [14]. A landmark study integrating plasma metabolomics with single-cell transcriptomics in COVID-19 patients demonstrated that each major immune cell type possesses a distinct metabolic signature and that disease severity correlates with cell-type-specific metabolic shifts, including increased glutathione metabolism as a compensatory response to oxidative stress [15]. The concept of using immune cell metabolomics as a disease biomarker is gaining traction, with recent proposals for real-time metabolic profiling of peripheral blood mononuclear cells as biomarkers for disease monitoring [16], and advances in single-cell spatial metabolomics enabling simultaneous profiling of metabolites and proteins in individual immune cells [17].

The search for blood-based biomarkers of pediatric OSAS has been active for nearly two decades. Shah and colleagues were pioneers in the field of serum proteins, identifying differentially regulated proteins capable of diagnosing OSAS with 93% sensitivity and 90% specificity [18]. Subsequent studies have explored plasma-based biomarker panels with moderate diagnostic performance [19,20], and a recent systematic review reported that serum-based biomarkers achieved a pooled sensitivity of 77.87% while urine-based biomarkers achieved 89.75% [21]. Despite these advances, no prior study has examined the metabolome of circulating immune cells in OSAS, either in adults or children, leaving a significant gap in our understanding of the intracellular metabolic reprogramming that drives immune dysfunction in this disease.

The present study addresses this gap by applying LC-MS-based metabolomics to PMNCs isolated from the peripheral blood of pediatric OSAS patients and healthy controls. This cell-specific approach constitutes a significant departure from conventional biofluid metabolomics and provides unique insights into the intracellular metabolic landscape of circulating immune cells under the presumed pathophysiological context of chronic intermittent hypoxia. We employed metabolite set enrichment analysis (MSEA) to identify enriched metabolic pathways and hierarchical clustering to characterize individual metabolite profiles, with the aim of exploring the immunometabolic mechanisms underlying pediatric OSAS by studying PMNCs metabolomics.

## 2. Results

### 2.1. Enrolled Population

The OSAS group included two female and five male pediatric patients who underwent adenotonsillectomy. Their ages ranged from 2 to 6 years. According to the Brodsky Scale, all children had tonsillar grades between III and IV, and were classified as ASA physical status I–III. These children experienced obstructive sleep apnea episodes lasting more than 10 s, occurring multiple times during the night. According to the institutional guideline, polysomnography was not performed for six out of seven of the OSAS group patients, as they all were >2 years and did not exhibit any of the following: obesity, Down syndrome, craniofacial abnormalities, neuromuscular disorders, sickle cell disease, or mucopolysaccharidoses [22]. All OSAS patients had BMI values within the normal range for age and sex, and none met criteria for overweight or obesity. Body Mass Index (BMI) values and the details of the ENT evaluation of the patients included in the study are shown in Table S1. As a routine standard of care, follow-up data were obtained for all these patients, showing a post-operative relevant improvement of symptoms.

The control group included five female and three male pediatric patients who came to the hospital for other reasons. Their ages ranged from 3 to 13 years.

Figure S2 shows the selection of the patients included in the metabolome study from the total cohort population.

### 2.2. Untargeted Metabolomics Analysis from PMNCs

All 15 PMNCs samples were subjected to LC-MS/MS. Mass spectrometry identified 96,869 features for positive ionization ESI+ and 75,469 features in negative-mode ESI−. Data were filtered based on the absence of MS2 and the lack of molecular names, as well as a delta mass of ±5 ppm. After filtering, 3801 (Supplementary File S1) metabolite annotations supported by MS/MS were retained. Multivariate analysis of the metabolomic datasets showed separation between OSAS and control samples in both ionization modes. In ESI+, PCA explained 41.7% of the total variance (PC1 28.2%, PC2 13.5%), and group differences were supported by PERMANOVA (F = 5.96, R^2^ = 0.31, *p* = 0.014). In ESI−, (Supplementary File S2) the first two principal components explained 50.8% of the variance (PC1 29.1%, PC2 21.7%), with PERMANOVA again confirming significant separation (F = 4.98, R^2^ = 0.27, *p* = 0.008). Cross-validation metrics (R^2^Y, Q) for PLS-DA models across increasing numbers of components are summarized in Table 1 (full cross-validation plots in Supplementary Figure S3). Permutation testing (1000 iterations) yielded a non-significant empirical *p*-value (*p* = 0.50) for the Q^2^ statistic in both modes, indicating that the PLS-DA predictive performance did not exceed chance.

Accordingly, PLS-DA was used only for exploratory visualization (Supplementary Figures S4 and S5), while PERMANOVA served as the primary statistical test for group separation.

**Figure 1:**
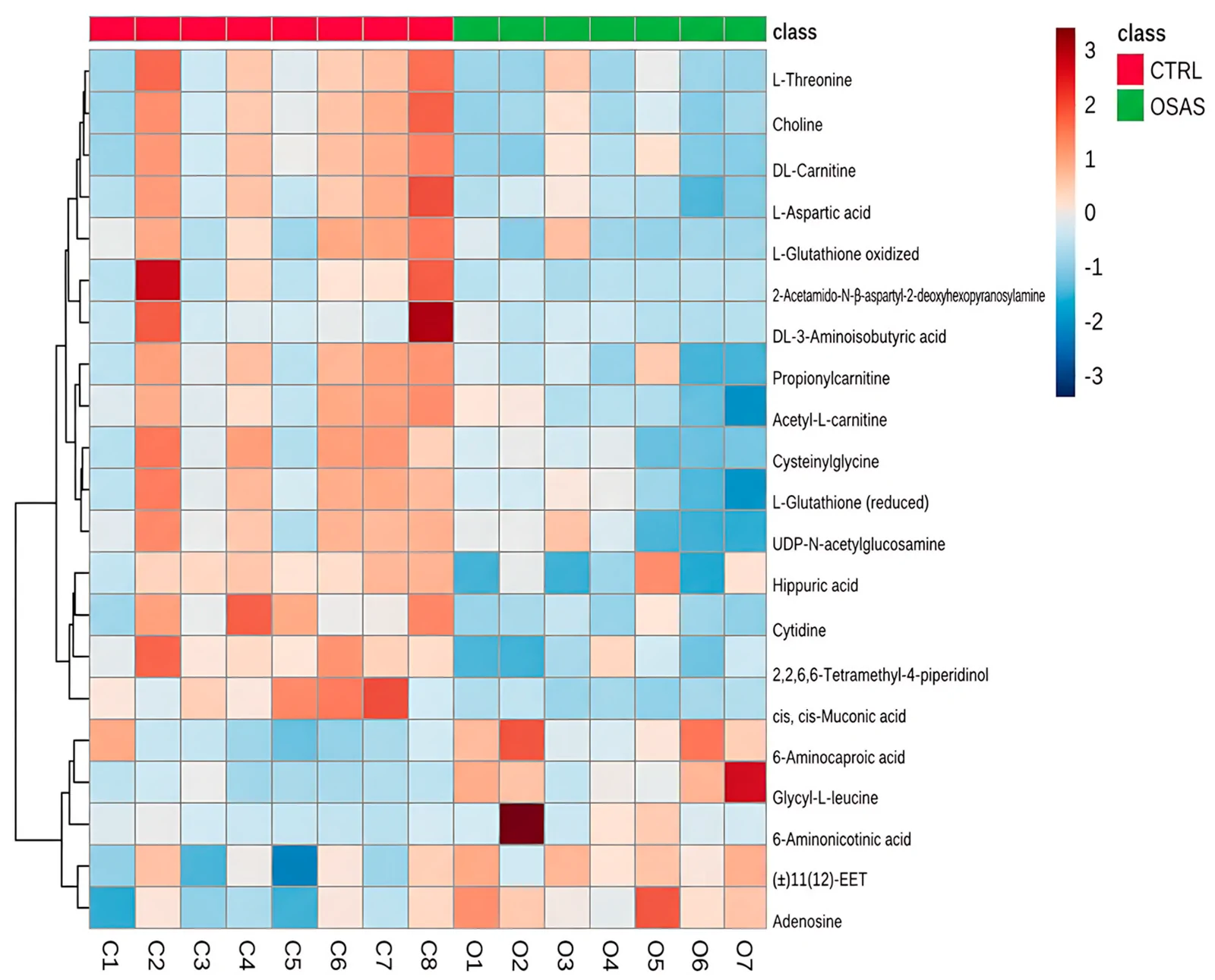
The heatmap illustrates the relative abundance profiles of the selected metabolites from the LC–MS/MS metabolomic analysis of the PMNCs samples. The columns represent the samples (controls (CTRL) in red and OSAS in green), and the rows represent the annotated metabolites. The color scale ranges from blue (low abundance) to red (high abundance). Hierarchical clustering (shown as dendrograms on the rows and columns) highlights the grouping of the samples based primarily on clinical group.

Only metabolites with an mzCloud Best Match score ≥70 in both ionization modes were included in downstream differential analyses. This filtering yielded 528 metabolites in ESI+ and 333 in ESI−, resulting in a common set of 311 annotated metabolites present in both modes. Univariate comparisons identified 21 nominally differentially abundant metabolites (Supplementary Files S3 and S4), visualized via hierarchical clustering in Figure 1. Upon Benjamini–Hochberg FDR correction (q < 0.05) was performed across all 2116 unique annotated metabolites included in the statistical analysis, only putatively annotated cis,cis-muconic acid retained statistical significance.

**Figure 2:**
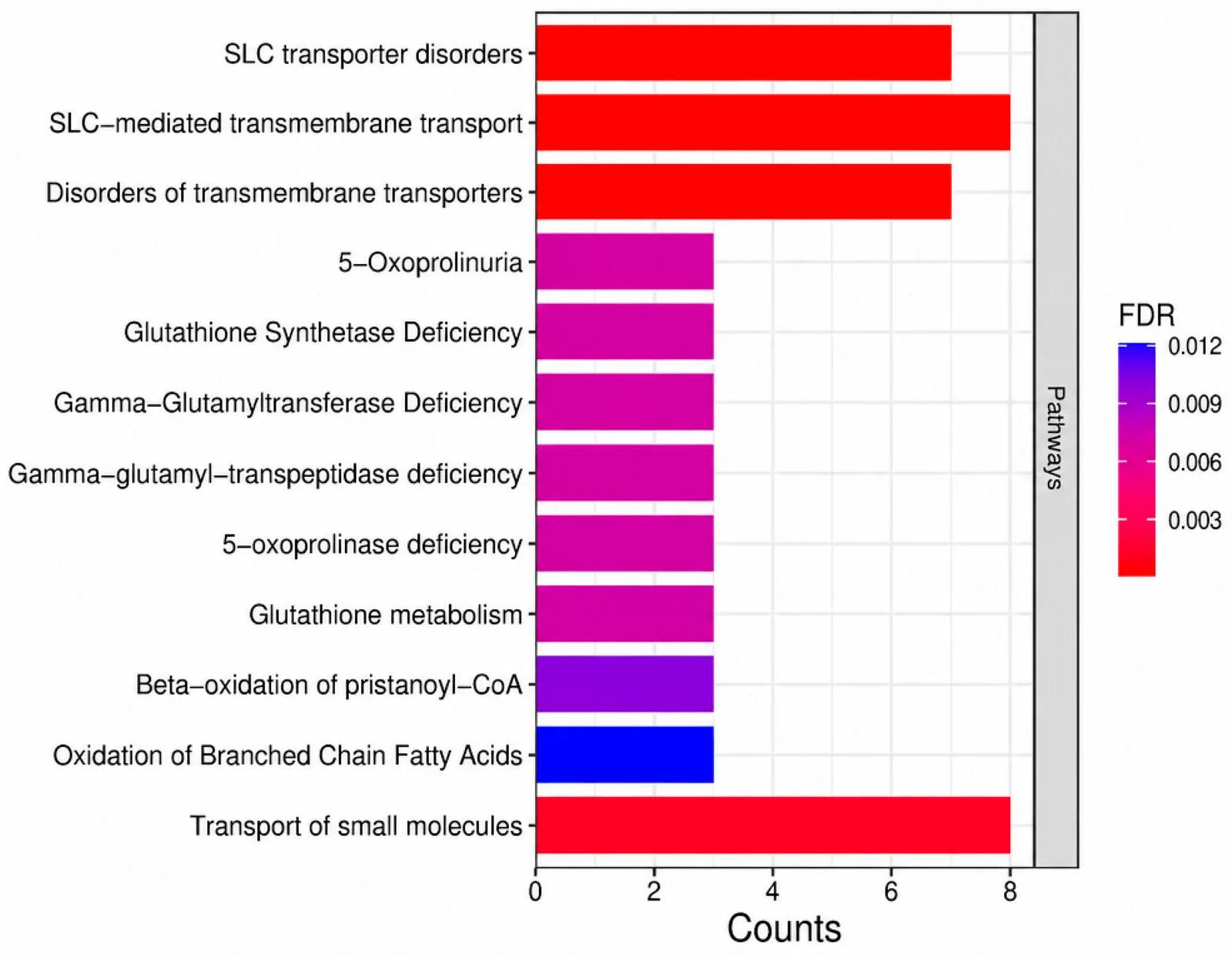
Metabolic set enrichment analysis of differentially abundant metabolites. The bar plot shows the enriched pathways ranked by the number of mapped metabolites. Bar color indicates BH-FDR-adjusted significance (red = lower FDR, blue = higher FDR). Overall, the figure is intended as an exploratory summary of pathway-level trends.

Two main abundance patterns were observed in the heatmap: a cluster of metabolites showing lower abundance in the OSAS group (including L-Threonine, Choline, 2-Acetamido-N-beta-aspartyl-2-deoxyhexopyranosylamine, and L-Aspartic acid) and a second cluster showing higher abundance in the OSAS group (including 6-Aminocaproic acid, Glycyl-L-leucine, and Adenosine). Sample clustering confirmed clear separation between controls and OSAS. Metabolite set enrichment analysis (MSEA) was performed on the 21 nominally significant metabolites using the MetaboAnalyst 6.0 Pathway Module. As these findings are based on uncorrected features, they represent exploratory pathway-level trends. As shown in Figure 2, pathways related to solute carrier (SLC) transporters, including SLC transporter disorders, SLC-mediated transmembrane transport, and disorders of transmembrane transporters, exhibited the strongest enrichment based on BH-FDR values. Pathways associated with glutathione metabolism (e.g., glutathione synthetase deficiency, gamma-glutamyltransferase deficiency, 5-oxoprolinuria) and mitochondrial lipid oxidation (beta-oxidation of pristanoyl-CoA, oxidation of branched-chain fatty acids) were also enriched. Overall, the enrichment profile points to potential alterations in transporter function, redox balance, and mitochondrial lipid pathways in OSAS PMNCs.

## 3. Discussion

The most significantly enriched pathways in the present analysis were SLC transporter disorders (FDR = 2.75 × 10^−5^), SLC-mediated transmembrane transport, and small molecule transport (Figure 2). In the context of OSAS, intermittent hypoxia stabilizes HIF-1α, reprogramming cellular metabolism from oxidative phosphorylation toward glycolysis and transactivating genes that encode glucose transporters (SLC2A/GLUT family), lactate transporters (MCT4/SLC16A3), and amino acid transporters [13]. Ryan et al. demonstrated that intermittent hypoxia selectively activates the NF-κB inflammatory pathway over the HIF-1α adaptive pathway in circulating leukocytes, promoting TNF-α production and neutrophil activation [14]. The enrichment of SLC transporter pathways in PMNCs of our OSAS cohort thus raises the possibility that metabolic rewiring occurs at the level of transporters, a phenomenon that was previously inferred only from transcriptomic and functional studies.

Seven significantly enriched pathways were found to be related to fatty acid metabolism. These include the transport of fatty acids; defective SLC27A4; the biosynthesis of unsaturated fatty acids; omega-9 fatty acid synthesis; free fatty acid receptors; carnitine-acylcarnitine translocase deficiency; and the beta-oxidation of branched-chain fatty acids (Figure 2). The enrichment of the carnitine-acylcarnitine translocase deficiency pathway suggests that there is impaired mitochondrial fatty acid oxidation (FAO) in PMNCs of OSAS patients.

Heatmap analysis of individual metabolites (Figure 1) may support these pathway-level findings. In the first metabolite block, concurrent depletion of DL-carnitine, acetyl-L-carnitine and propionylcarnitine in OSAS PMNCs raises the possibility that β-oxidation is impaired. Furthermore, the acetylcarnitine-to-propionylcarnitine ratio has been causally associated with insomnia risk via Mendelian randomization [23], and their depletion specifically within PMNCs suggests that immune cells experience a direct bioenergetic deficit under chronic intermittent hypoxia [24]. Chronic intermittent hypoxia in OSAS activates HIF-1α, which suppresses mitochondrial fatty acid β-oxidation and favors intracellular lipid accumulation rather than channelling free fatty acids into energy production [25]. These specific fatty acids carry important immunomodulatory implications. Lauric acid promotes Th17 cell differentiation and stimulates macrophage interleukin-12 secretion, myristic acid increases monocyte chemoattractant protein-1 expression and reduces macrophage phagocytic capacity [26,27]. Oleic acid, at elevated intracellular concentrations, can promote Th17 differentiation by binding to the IL-17a promoter via RORγt [28]. Fatty acid metabolism is now recognized as a fundamental determinant of T cell fate. Effector T cells rely on de novo fatty acid synthesis, whereas regulatory T cells depend on FAO for their suppressive function [29,30]. FFAR4 activation on T cells reduces pro-inflammatory cytokine production and promotes regulatory function [31]. The bidirectional pattern of carnitine depletion and pro-inflammatory fatty acid accumulation may directly contribute to the Th17/Treg imbalance that is consistently documented in OSAS. In this condition, Th17 frequencies are markedly elevated, RORγt expression is increased, and the Th17/Treg ratio positively correlates with disease severity [3].

Seven pathways related to glutathione metabolism were significantly enriched, including enzymes of the γ-glutamyl cycle (5-oxoprolinase, γ-glutamyltranspeptidase, and glutathione synthetase). This could plausibly reflect stress affecting the entire reduced glutathione (GSH) turnover machinery in OSAS PMNCs.

Peripheral lymphocytes from OSAS patients exhibit significantly decreased GSH levels, increased oxidised glutathione (GSSG) levels, and a reduced GSH/GSSG ratio [4,32]. The pathway-level narrative (transporter function, glutathione metabolism, fatty acid oxidation) is offered as a hypothesis-generating biological framework for future targeted validation, not as confirmed findings.

Heatmap analysis provided individual-metabolite resolution to these pathway findings. Reduced glutathione, oxidized glutathione, and cysteinylglycine were simultaneously depleted in OSAS PMNCs. Cysteinylglycine is the immediate degradation product of glutathione via γ-glutamyltranspeptidase, and the cysteinylglycine-to-glutathione ratio has been proposed as a surrogate marker of oxidative stress burden [33]. The depletion of both GSH and GSSG, along with their degradation product, suggests that the entire glutathione pool is consumed and not adequately replenished, demonstrating that the oxidative stress burden in pediatric OSAS is experienced directly by immune cells.

Several additional metabolites depleted in OSAS PMNCs provide further mechanistic context. Uridine-5′-di-phospho-N-acetylglucosamine (UDP-GlcNAc), the essential substrate for N- and O-linked glycosylation, was reduced in our study. O-GlcNAcylation favours the differentiation of T regulatory cells over the generation of pro-inflammatory T helper Th1 and Th17, suggesting that its depletion could contribute to the Th17/Treg imbalance [34]. Choline, which protects against chronic intermittent hypoxia-induced vascular injury through activation of α7 non-neuronal nicotinic acetylcholine receptors [35], was similarly diminished. Hippuric acid, a gut microbial co-metabolite of aromatic amino acids [36], was decreased in our analysis, consistent with documented gut dysbiosis in OSAS. The depletion of cytidine and 2′-deoxycytidine further support the enrichment of pyrimidine transport pathways and likely reflects increased nucleotide demand in activated immune cells.

Three enriched pathways relate to incretin biology (Figure 2): glucagon-like peptide-1 (GLP-1) receptor, glucose-dependent insulinotropic peptide (GIP) receptor, and incretin synthesis/inactivation. In pediatric OSAS, metabolic consequences include altered glucose metabolism, impaired insulin secretion, and elevated branched-chain amino acids [37]. This finding is particularly timely given the FDA approval of tirzepatide for adult OSAS based on the SURMOUNT-OSA trial study, which demonstrated significant reductions in AHI, body weight, hsCRP, and hypoxic burden [38]. GLP-1 receptors are expressed on immune cells, where agonists exert anti-inflammatory effects by reducing NF-κB signaling [39]. The enrichment of incretin pathway metabolites within PMNCs provides a molecular rationale for the potential efficacy of incretin-based therapies in pediatric OSAS.

Five pathways related to neurotransmitter biology reached significance (Figure 2). OSAS patients exhibit lower glutamate and γ-aminobutyric acid (GABA) and higher glutamate levels in the insular cortex, contributing to autonomic dysregulation [40,41]. Immune cells express glutamate receptors and the enzymatic machinery necessary for neurotransmitter metabolism. Glutamate serves as a precursor for GSH synthesis, linking this cluster to the glutathione pathways, and as a TCA cycle intermediate via α-ketoglutarate. The enrichment of glutamate metabolism in PMNCs reflects the convergence of oxidative stress, metabolic reprogramming, and immunomodulatory signaling, a finding unique to our cell-specific metabolomic approach.

The metabolic pathways identified in this exploratory study may provide insight into biological mechanisms that could be relevant for future therapeutic research. However, any potential translational implications remain hypothesis-generating and should not be interpreted as evidence supporting clinical interventions. The enrichment of γ-glutamyl cycle pathways is consistent with previous studies implicating oxidative stress in OSAS and may support further investigation of glutathione-targeted strategies, such as N-acetylcysteine [42,43]. Likewise, the alterations observed in carnitine-related and fatty acid oxidation pathways are in line with mechanisms that have been proposed to influence mitochondrial function and immune cell bioenergetics, providing a rationale for future studies evaluating interventions such as L-carnitine or omega-9 fatty acid supplementation [44]. Nevertheless, these considerations are intended solely to place our pathway-level findings within the context of the existing literature. Given the limited sample size of this pilot study and the exploratory nature of the metabolomic analysis, dedicated mechanistic and adequately powered interventional studies will be required before any therapeutic implications can be established.

Several limitations of this study should be acknowledged, foremost among them the limited sample size (*n* = 15; 8 controls and 7 OSAS patients). Untargeted metabolomic profiles are inherently influenced by substantial inter-individual biological variability, including differences in diet, circadian rhythm, pubertal stage, medication exposure, and gut microbiome composition, factors that could not be fully controlled in a cohort of this size. Consequently, some of the observed metabolic differences may reflect cohort-specific variability rather than broadly generalizable features of pediatric OSAS.

The lack of detailed clinical severity data, such as AHI and oxygen desaturation nadir, limits the possibility of directly linking the observed metabolic alterations to disease severity in this pilot cohort. Larger, deeply phenotyped studies will be required to determine whether these metabolic changes track with clinical burden and to clarify their potential translational relevance.

However, following Benjamini–Hochberg correction for multiple testing, only putatively annotated cis,cis-muconic acid remained statistically significant (FDR-adjusted q < 0.05). Likewise, permutation testing of the PLS-DA models (1000 iterations) yielded non-significant empirical *p*-values (*p* = 0.50) in both ionization modes, indicating that the apparent predictive performance of the supervised models was not greater than expected by chance in a dataset of this size and dimensionality. Accordingly, PLS-DA was used exclusively as an exploratory visualization approach and not for predictive or diagnostic purposes.

Accordingly, the metabolic alterations described in this study, including changes related to transmembrane transport, glutathione metabolism, fatty acid oxidation, and neurotransmitter/incretin signaling, can be interpreted as hypothesis-generating observations that provide a biological framework for future investigation rather than definitive evidence of disease-specific metabolic signatures. In contrast, the overall metabolic differences between groups were supported by PERMANOVA, a non-parametric, distance-based method that is less susceptible to overfitting than supervised classification approaches and demonstrated significant group separation in both ionization modes (ESI+: F = 5.96, R^2^ = 0.31, *p* = 0.014; ESI−: F = 4.98, R^2^ = 0.27, *p* = 0.008).

Another limitation of this pilot study is the age discrepancy between the OSAS group (2–6 years) and the control group (3–13 years). In pediatric populations, developmental changes across this age span can influence systemic metabolism. To evaluate whether age acted as a major confounder for our primary finding, we performed an exploratory Spearman correlation analysis between chronological age and cis,cis-muconic acid levels (Supplementary Figure S6). The analysis yielded a positive correlation coefficient of moderate magnitude (rho = 0.39). Although this relationship did not reach formal statistical significance (*p* = 0.15), the small sample size of our cohort (*n* = 15) severely limits statistical power and increases the risk of a Type II error. Consequently, we cannot definitively rule out that the age imbalance may have partially contributed to the observed differences in metabolite abundance. Future studies incorporating larger, age-matched pediatric cohorts are essential to determine the exact extent to which these metabolic shifts are driven by OSAS pathophysiology rather than developmental trajectories.

Sex distribution represents an additional potential source of confounding in this study. The OSAS group included 5 males and 2 females, whereas the control group included 3 males and 5 females. Although this difference was not statistically significant (Fisher’s exact test, *p* = 0.32), the limited sample size reduces the statistical power to detect modest imbalances between groups. As an exploratory analysis, we evaluated the relationship between sex and the abundance of putatively annotated cis,cis-muconic acid. No statistically significant difference in metabolite intensity was observed between males and females (Mann–Whitney test, *p* = 0.54). While these findings do not provide evidence of a statistically significant sex effect within this cohort, the limited statistical power of this pilot study does not allow residual sex-related confounding to be excluded. Consequently, future studies involving larger, sex-balanced cohorts will be necessary to distinguish disease-associated metabolic alterations from potential sex-related metabolic variation and to confirm the reproducibility of the present findings.

An additional limitation concerns the pathway enrichment analysis, which was performed using a panel of only 21 annotated metabolites. Although all reported pathways remained statistically significant following pathway-level FDR correction (hypergeometric over-representation analysis against the RaMP-DB reference database), the robustness of the enrichment results is inherently dependent on the size and composition of the input metabolite list. Consequently, these findings can be interpreted as exploratory and hypothesis-generating rather than as definitive evidence of pathway dysregulation. These considerations, together with the exploratory nature of the underlying metabolomic dataset, warrant cautious interpretation of the pathway-level findings. Future studies involving larger, independent cohorts and targeted quantitative validation will be necessary to determine whether the biological pathways highlighted here represent reproducible features of pediatric OSAS.

However, some points strength should also be considered. To the best of our knowledge, this is the first study targeting the metabolomic profile of PMNCs of pediatric patients with OSAS. These findings suggested the possible implication of several mechanisms in the pathogenesis of OSAS, supporting the proof of concept recognition that OSAS is a systemic inflammatory-metabolic disease with molecular consequences in circulating immune cells. In fact, previous blood-based biomarker approaches have achieved pooled sensitivities of 77–90% using serum proteomics or urine-based biomarkers [18,21].

Finally, the observed pattern of metabolic disruption, encompassing carnitine depletion, glutathione exhaustion, altered incretin signalling, and fatty acid accumulation, raises the hypothesis that pediatric OSAS may represent, at least in a subset of patients, an age-dependent early manifestation of metabolic syndrome.

**Figure 3:**
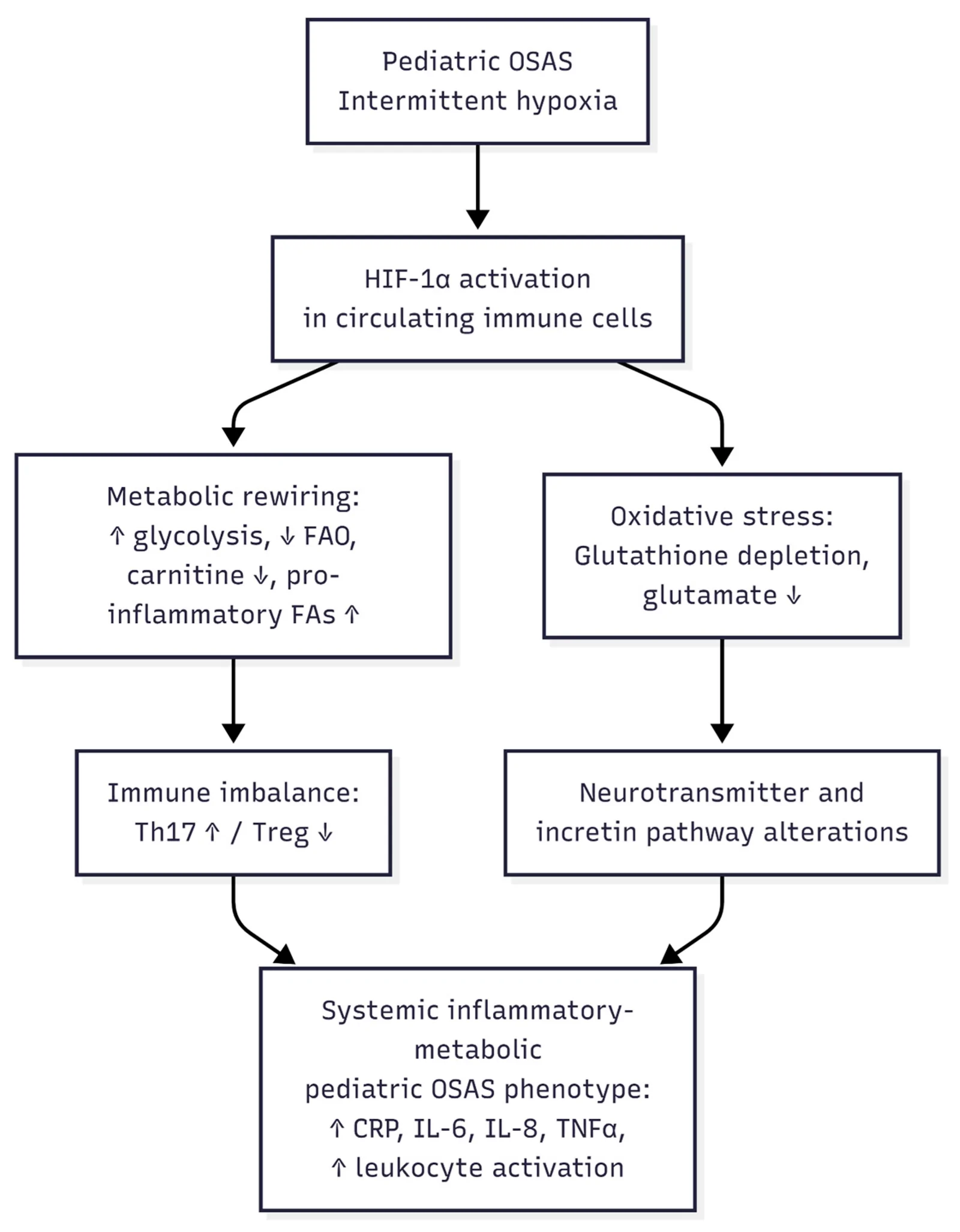
Proposed literature-based conceptual model of the putative metabolomic signature in pediatric OSAS. According to mechanisms reported in the literature, intermittent hypoxia in pediatric obstructive sleep apnea syndrome may activate HIF-1α in circulating immune cells, potentially inducing metabolic rewiring toward glycolysis, impaired mitochondrial fatty acid oxidation, carnitine depletion, and accumulation of pro-inflammatory fatty acids. This metabolic state, together with the glutathione depletion and reduced glutamate availability observed as exploratory associations in the present pilot cohort, is hypothesized to contribute to Th17/Treg imbalance, neurotransmitter and incretin pathway perturbations, and a systemic inflammatory-metabolic phenotype characterized by elevated CRP, IL-6, IL-8, and TNF-α, consistent with previous reports [2,19]. This schematic integrates established mechanisms from the cited literature with exploratory findings from the present study and should be interpreted as a hypothesis-generating framework rather than a pathway experimentally validated by our data.

Figure 3 presents a literature-based conceptual model integrating established mechanisms from the cited literature with the exploratory findings of this pilot study.

It should be emphasized that after correction for the large number of tests inherent to untargeted metabolomics (Benjamini–Hochberg FDR), only putatively annotated cis,cis-muconic acid retained statistical significance (q < 0.05), whereas the remaining metabolites discussed above should be regarded as nominally significant trends rather than validated biomarkers. Consistent with the exploratory and hypothesis-generating nature of this pilot study, our aim was not to establish diagnostic biomarkers but to describe plausible pathophysiological signatures—transporter dysfunction, redox imbalance, and impaired fatty acid oxidation—that warrant confirmation through targeted, adequately powered studies.

## 4. Materials and Methods

### 4.1. Patient Enrolment

Patients diagnosed with OSAS were enrolled at the surgical unit of the Institute for Maternal and Child Health—IRCCS “Burlo Garofolo” (Trieste, Italy) during 2024 and 2025. The pateints included in the study were part of a cohort population of pediatric pateints undergoing tonsillectomy for other indications as well such as PFAPA (periodic fevers with aphthous stomatitis, pharyngitis, and adenitis) and recurrent fevers.

Written informed consent was obtained from all participants in accordance with institutional ethical guidelines.

Inclusion criteria for OSAS:

- Age (2–13).

Symptoms of OSAS (apnea, heavy snoring, sleep disturbance) confirmed by video recording.

- Tonsil hypertrophy 3 or 4 according to the Brodsky scale.

Exclusion criteria for OSAS:

- Craniofacial abnormality.
- Neuromuscular disease.
- Chromosomal abnormality.
- Previous adenotonsillar surgery.
- Bleeding disorder.
- Cardiopulmonary disease.
- History of recurrent tonsillitis.

The control group consisted of anamnestic healthy patients with age (2–13) selected on the ground of no history of apnoea or obesity. Exclusion criteria for the control group also included the presence of autoimmune disease, immunodeficiency, growth hormone deficiency, or inflammatory bowel disease.

### 4.2. Sample Collection

Blood samples were collected from patients who received diagnoses of OSAS at the surgical unit. The samples were kept at 4 °C and processed within three hours of collection. In addition, blood samples from healthy volunteers were obtained to serve as controls.

### 4.3. Isolation of Peripheral Blood Mononuclear Cells (PMNCs)

PMNCs were isolated from peripheral blood using a density gradient method. Briefly, using a 15 mL tube, 4 mL of whole blood was layered onto 2 mL of gradient solution Lympholyte (DVCL5015R, Cedarlane Laboratories, Burlington, ON, Canada) and centrifuged at 700× *g* for 40 min at room temperature without brake. Following centrifugation, the white cell layer appearing as a distinct buffy interface was carefully collected. The isolated cells were then resuspended in freezing medium composed of 90% fetal bovine serum (FBS) and 10% dimethyl sulfoxide (DMSO; EMR031100, Euroclone S.p.A., Pero, Italy) and stored at −80 °C until further use.

### 4.4. Extraction of Polar Metabolites Using 80% Methanol

Frozen PMNCs were thawed and centrifuged at 300× *g* for 5 min at 4 °C to obtain a pellet. The cells were washed with 0.9% NaCl solution and centrifuged again under the same conditions to remove residual debris and the solution was aspirated. Metabolic activity was quenched by the immediate addition of 1 mL of ice-cold 80% methanol/20% water (*v*/*v*), pre-equilibrated at −80 °C, to each sample. Samples were then incubated at −80 °C for 30 min to ensure complete quenching and protein precipitation. After incubation, samples were vortexed for 20 s and subsequently centrifuged at 16,000× *g* for 10 min at 4 °C to pellet cellular debris and precipitated proteins. The resulting supernatants were carefully transferred to fresh tubes and dried under vacuum using a SpeedVac concentrator (Eppendorf SE, Hamburg, Germany) without heat application to prevent metabolite degradation. Dried extracts were stored at −80 °C until further analysis. Protein quantification was performed with the Bradford assay. Before LC-MS/MS analysis, dried extracts were reconstituted in 90% acetonitrile/10% water (*v*/*v*).

### 4.5. PMNCs Polar Metabolome Analysis

The polar metabolome was analyzed using a Vanquish Neo UHPLC system (Thermo Fisher Scientific, Bremen, Germany) coupled to an Orbitrap Exploris 240 mass spectrometer (Thermo Fisher Scientific). Prior to analysis, the mass spectrometer was calibrated in both positive and negative ionization modes using Thermo Pierce™ FlexMix calibration solutions. Chromatographic separation was achieved on a SeQuant^®^ ZIC^®^-HILIC column (Sigma-Aldrich, St. Louis, MO, USA). (150 × 2.1 mm, 3.5 µm, 100 Å) maintained at 60 °C, with a flow rate of 80 µL/min. Data acquisition was performed in full MS scan mode over a mass range of 80–1000 *m*/*z*, with data-dependent MS/MS (dd-MS^2^) fragmentation. Mass resolution was set to 45,000 FWHM for full MS and 15,000 FWHM for MS/MS scans. The precursor isolation window was set to 1.6 *m*/*z*, and fragmentation was performed using normalized collision energies (NCE) of 10, 20, and 30. The auxiliary gas heater temperature was maintained at 300 °C. To monitor instrument stability throughout the acquisition, a pooled quality control (QC) sample was prepared by combining equal aliquots from all individual sample extracts. 

Pooled quality control (QC) samples were injected at regular intervals throughout the LC-MS acquisition sequence (*n* = 3 for ESI+ and *n* = 3 for ESI−) to monitor analytical performance, and the injection order of biological samples was randomized across the CTRL and OSAS groups. No post-acquisition signal drift correction was applied. Analytical reproducibility was evaluated by calculating the coefficient of variation (CV) across pooled QC injections for putatively annotated cis,cis-muconic acid, the only metabolite remaining statistically significant after FDR correction. The resulting CV was 17.0%, indicating satisfactory analytical reproducibility. PCA score plots including pooled QC samples are provided in Supplementary Figure S1.

Data processing was performed using Compound Discoverer software (version 3.3; Thermo Fisher Scientific). Chromatographic alignment was carried out using an adaptive curve alignment algorithm implemented within the software. Feature detection was performed using the following parameters: a mass tolerance of 5 ppm, a retention time tolerance of 0.2 min, a minimum peak intensity threshold of 100,000 arbitrary units (a.u.), and a signal-to-noise ratio threshold of 5. Compound identification was achieved by spectral matching against the Human Metabolome Database (HMDB). Data were normalized using median normalization before downstream statistical analysis.

### 4.6. Statistical and Enrichment Analysis

Statistical and multivariate analyses were performed using the MetaboAnalyst 6.0 platform. Before statistical analysis, a low-variance filter was applied using the default implementation in MetaboAnalyst 6.0, employing the standard deviation (SD) as the variance measure and removing the lowest 40% of features based on variance across samples.

Data were subsequently log_2_-transformed and mean-centered using autoscaling to ensure homogeneity of variance and comparability across metabolites. Multivariate patterns were explored using two complementary approaches: Principal Component Analysis (PCA), and supervised Partial Least Squares Discriminant Analysis (PLS-DA). PLS-DA models were cross-validated using a leave-one-out (LOOCV) approach, evaluating both full five-component and reduced two-component models for each ionization mode. The optimal number of latent components was selected based on the maximum Q^2^ value (ESI+: component 3, Q^2^ = 0.73; ESI−: component 4, Q^2^ = 0.67 for the five-component models; component 2, Q^2^ = 0.61 for both ionization modes in the reduced models). To assess whether the observed predictive performance exceeded that expected by chance given the high dimensionality of the dataset, a permutation test (1000 iterations) was performed by randomly permuting the class labels. The empirical *p*-value for the Q^2^ statistic was 0.50 for both ionization modes, indicating that the predictive performance of the PLS-DA models was not significantly greater than expected under random class assignments. Accordingly, PLS-DA was used solely for exploratory visualization rather than predictive modeling and was not employed to support classification, prediction, or biomarker discovery claims.

Statistical evidence for overall metabolic separation between groups was instead evaluated using Permutational Multivariate Analysis of Variance (PERMANOVA, Euclidean distance, 999 permutations), a non-parametric, distance-based hypothesis test that evaluates group differences without fitting a supervised classification model and is therefore not susceptible to model overfitting.

Differential abundance between the OSAS group and controls was assessed by combining a fold change (FC) threshold (FC ≥ 1.5 or FC ≤ 0.67) with a non-parametric Mann–Whitney U test.

Mann–Whitney U test *p*-values were computed using MetaboAnalyst 6.0’s standard univariate statistics implementation. To control the false discovery rate (FDR) arising from the large number of features tested in untargeted metabolomics, raw *p*-values were subsequently adjusted using the Benjamini–Hochberg (BH) procedure. Only metabolites with an FDR-adjusted q-value < 0.05 were considered statistically significant; all other nominally significant findings (raw *p* < 0.05) are reported as exploratory, hypothesis-generating trends. The resulting nominally differentially abundant metabolites were visualized using hierarchical clustering heatmaps.

Metabolite set enrichment analysis was carried out using the Enrichment Analysis module implemented in MetaboAnalyst 6.0, with pathway enrichment performed against the RaMP-DB (Relational Database of Metabolomics Pathways).

**Figure 4:**
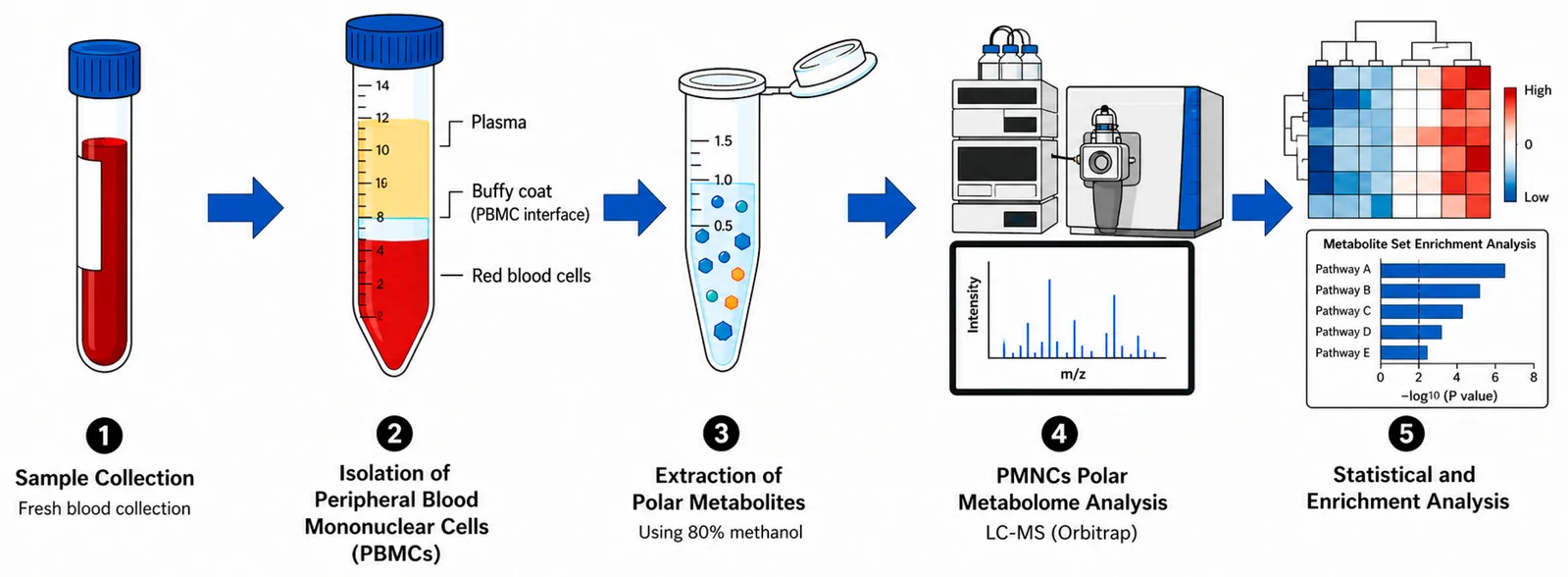
Workflow of the study.

The workflow of the study is illustrated in Figure 4.

## 5. Conclusions

This pilot study supports the application of cell-specific metabolomics in peripheral blood mononuclear cells to investigate the pathophysiology of pediatric OSAS at the level of circulating immune cells. Multivariate analyses, together with pathway enrichment analysis, revealed disease-associated metabolic alterations involving mitochondrial fatty acid oxidation, glutathione metabolism, and transmembrane transport, consistent with the inflammatory and metabolic dysregulation previously described in pediatric OSAS.

However, these findings should be interpreted in light of the exploratory nature of the study and its limited sample size (*n* = 15; 7 OSAS and 8 controls), which limits the statistical power and the generalizability of the observed associations. Following correction for multiple testing across the untargeted metabolomic dataset, only putatively annotated cis,cis-muconic acid remained statistically significant (FDR-adjusted q < 0.05), whereas the remaining metabolites should be regarded as exploratory observations contributing to the interpretation of altered metabolic pathways rather than definitive individual associations. Likewise, permutation testing indicated that the predictive performance of the PLS-DA models was not significantly greater than expected by chance, supporting their use as exploratory visualization tools rather than predictive or classification models.

Accordingly, the present findings should be considered hypothesis-generating. They do not support, at this stage, claims of diagnostic utility or therapeutic relevance. Overall, this study provides proof-of-concept evidence that pediatric OSAS is associated with metabolic reprogramming of circulating immune cells and highlights biological pathways, including fatty acid oxidation, glutathione metabolism, and transmembrane transport, that warrant further investigation. Because polysomnographic and objective hypoxia data were unavailable for most participants, the causal role of intermittent hypoxia in the observed metabolic alterations cannot be established, and these findings should be interpreted as exploratory and hypothesis-generating. Future studies involving larger, independent, age-matched, and longitudinal cohorts, ideally complemented by targeted quantitative metabolomic analyses, will be essential to validate these pathway-level alterations and determine whether they represent reproducible mechanisms underlying pediatric OSAS.

## Figures and Tables

**Table 1:** Leave-one-out cross-validation (LOOCV) performance of PLS-DA models for the ESI+ and ESI− metabolomic datasets. For each ionization mode, both a full five-component model and a reduced two-component model were evaluated. The optimal number of latent components was selected based on the maximum Q^2^ value. Model performance is reported in terms of classification accuracy, R^2^Y (goodness of fit), and Q^2^ (predictive ability) for the optimal component of each model. Although the models achieved relatively high Q^2^ values (0.61–0.73), permutation testing (1000 iterations) yielded a non-significant empirical *p*-value (*p* = 0.50) for Q^2^ in both ionization modes. These results indicate that the observed predictive performance was not significantly greater than expected by chance, likely reflecting the limited sample size (*n* = 15) relative to the high dimensionality of the metabolomic data. Consequently, PLS-DA was used solely as an exploratory visualization method, whereas statistical evidence for group separation was based on the PERMANOVA analysis.

Model	Optimal Component	Accuracy	R^2^Y	Q^2^
ESI+ (5 comp.)	Component 3	~1.00	~1.00	**0.73**
ESI+ (2 comp.)	Component 2	~0.93	~0.96	**0.61**
ESI− (5 comp.)	Component 4	~0.90	~1.00	**0.67**
ESI− (2 comp.)	Component 2	~0.93	~0.96	**0.61**

## Data Availability

The original contributions presented in this study are included in the article and Supplementary Materials. Further inquiries can be directed to the corresponding authors.

## Supplementary Materials

The following supporting information can be downloaded at: https://www.mdpi.com/article/10.3390/ijms27177658/s1.
