# Supplementary material for: Peripheral Mononuclear Cells Metabolomics in Obstructive Sleep Apnea Syndrome (OSAS): An Exploratory Pilot Study of Immunometabolic Signatures
Source: Int J Mol Sci. 2026 Aug 26;27(17):7658. doi: 10.3390/ijms27177658 (PMC13566245; doi:10.3390/ijms27177658)
Supplement: Supplementary file 1 [file ijms-27-07658-s001.zip › Supplementary figures.pdf]

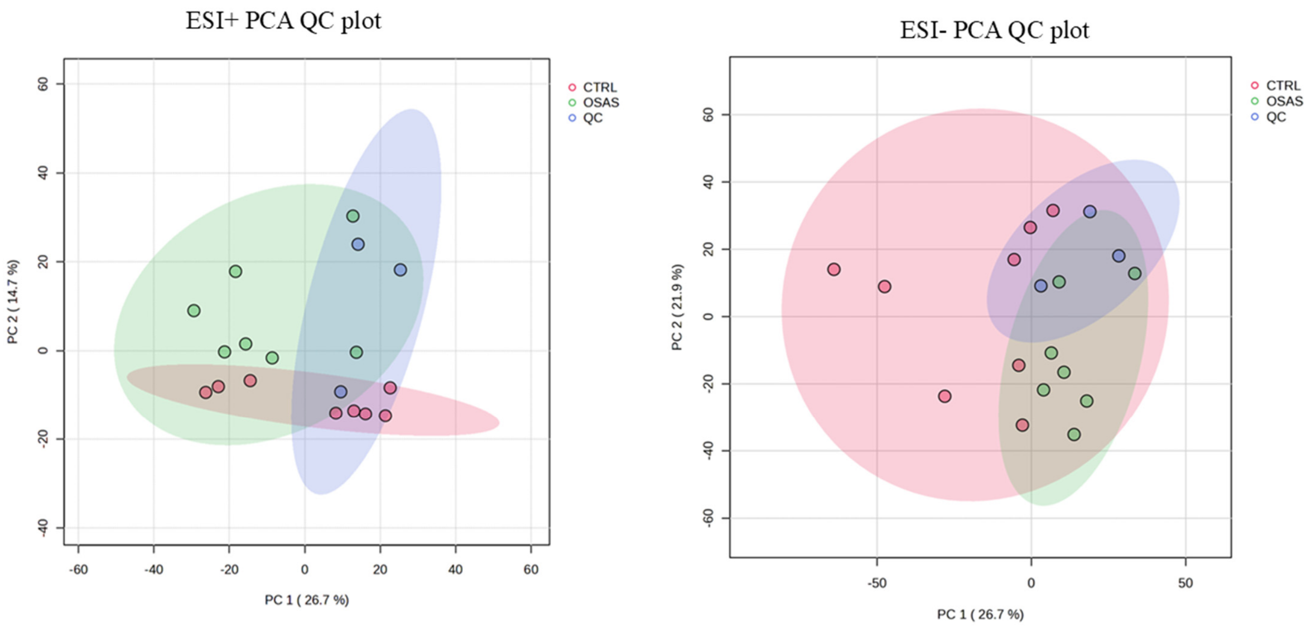

Supplementary Figure S1. Principal component analysis (PCA) score plots including pooled quality control (QC) samples (blue), CTRL samples (red), and OSAS samples (green) for the ESI+ (left) and ESI− (right) datasets. QC samples (n = 3 per ionization mode), injected at regular intervals throughout the analytical sequence, were consistently positioned within the overall multivariate space and showed no evidence of analytical outliers, supporting the overall stability and reproducibility of the LC-MS analytical platform during data acquisition. The coefficient of variation (CV) calculated across QC injections for **cis,cis-muconic acid**, the only metabolite remaining statistically significant after FDR correction, was **17.0%**, consistent with satisfactory analytical reproducibility.

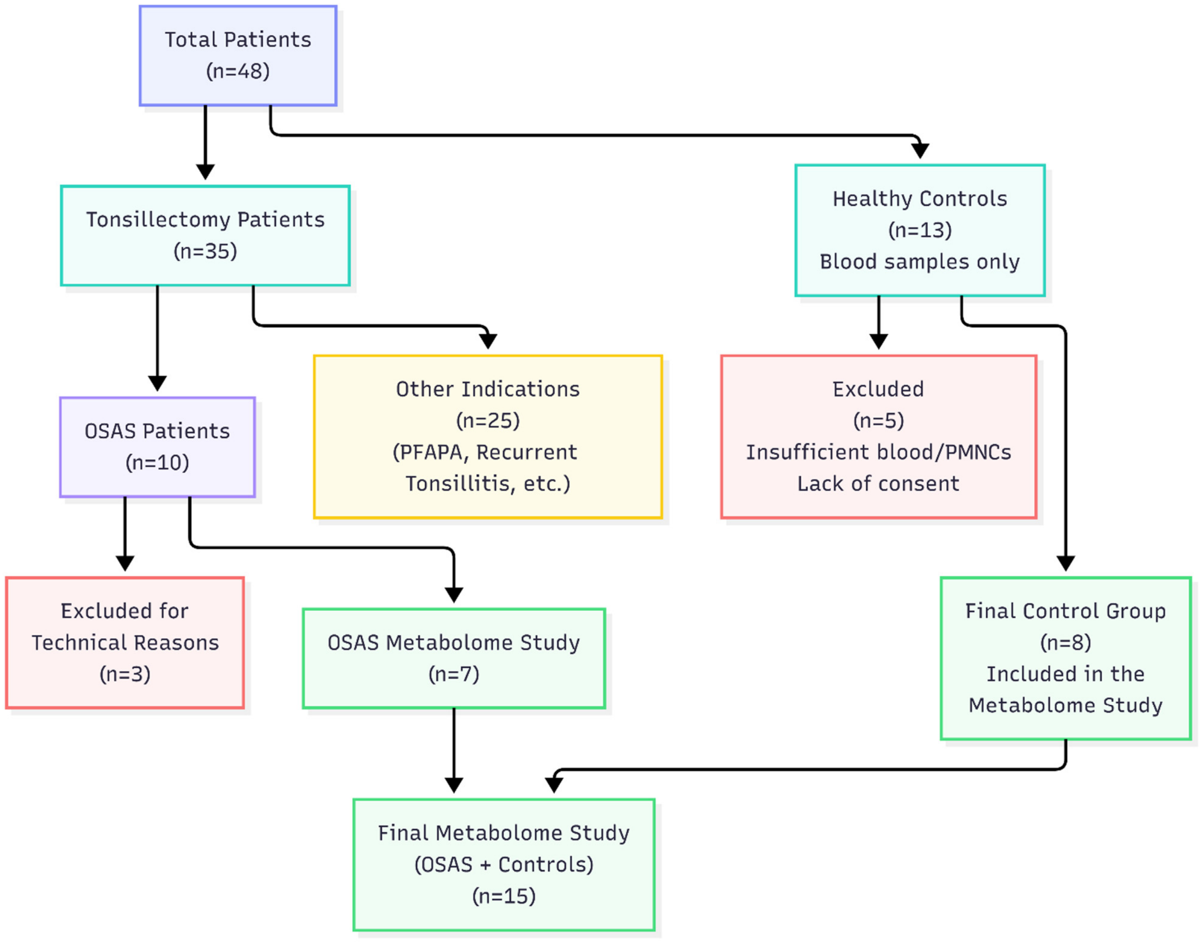

Supplementary Figure S2. Patient recruitment and selection flow diagram. Of 35 peditaric patints subjected to tonsillectomy for OSAS and other reasons like PFAPA and recurrent tonsillitis, 10 pateints met the inclusion criteria of OSAS, among them 3 were excluded for technial reasons (insufficient blood/PMNCs). On the other hand, only blood samples were collected from 13 healthy children to serve as control group of the study, among them 5 were excluded for technical reasons or lack of consent. The final metabolome study included 15 samples (OSAS =7, Controls=8).

ESI+ cross-validation plots

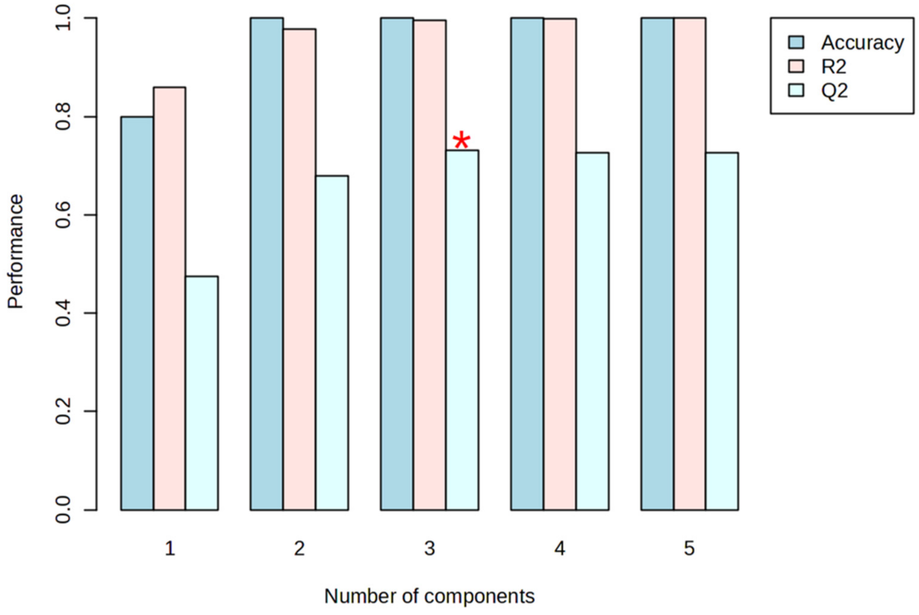

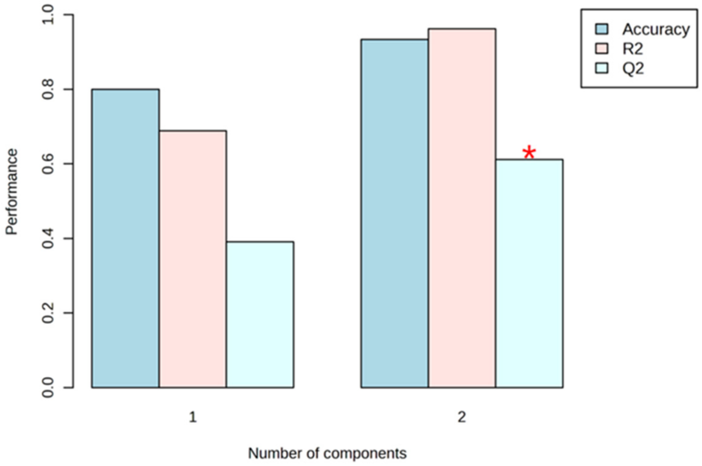

ESI- cross-validation plots

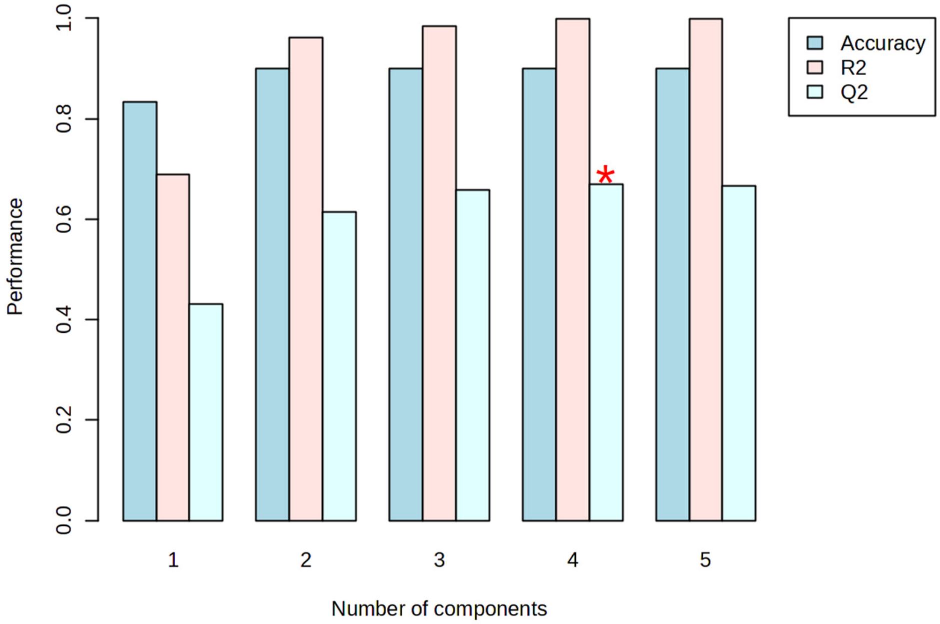

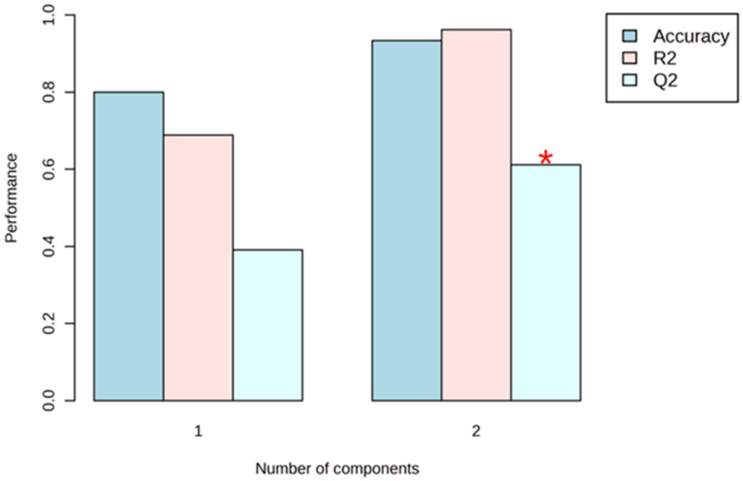

Supplementary Figure S3. Leave-one-out cross-validation (LOOCV) performance of the PLS-DA models for the ESI+ and ESI− metabolomic datasets, shown separately for the five-component and two-component models. Classification accuracy, R^2Y, and Q^2 are reported as a function of the number of latent components. The optimal model complexity (red asterisk) was determined based on the maximum Q^2 value, corresponding to component 3 for the ESI+ five-component model ($Q^2 = 0.73$), component 2 for the ESI+ two-component model ($Q^2 = 0.61$), component 4 for the ESI− five-

component model ($Q^2 = 0.67$), and component 2 for the ESI− two-component model ($Q^2 = 0.61$). Model significance was further assessed by permutation testing (1,000 iterations), which yielded an empirical p-value of 0.50 for the Q^2 statistic in both ionization modes. This result indicates that the observed predictive performance was not significantly different from that expected by chance, likely reflecting the limited sample size ($n = 15$) relative to the high dimensionality of the metabolomic dataset. Accordingly, PLS-DA was used exclusively as an exploratory visualization tool rather than for predictive modeling, as described in the Methods and Results sections.

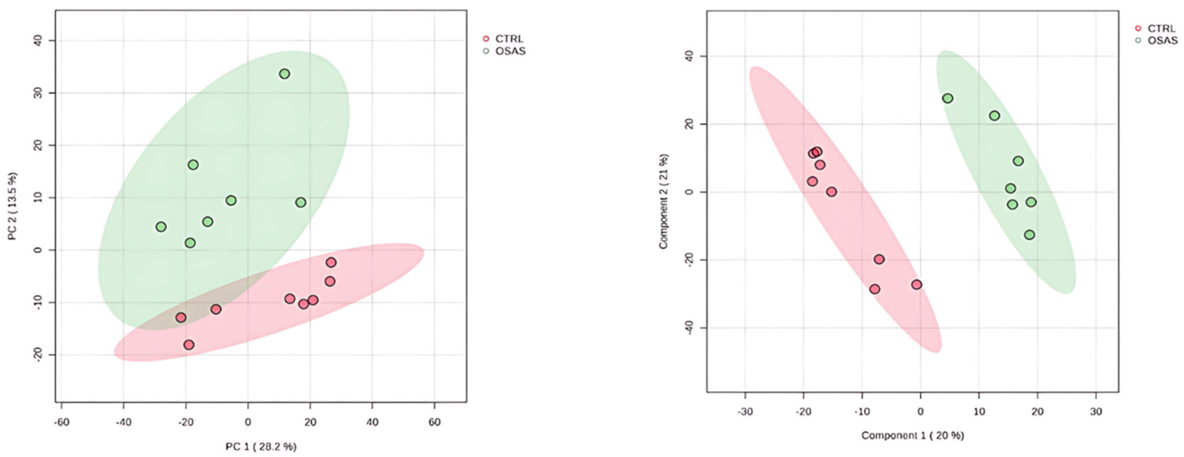

Supplementary Figure S4. Multivariate analysis of positive-ion metabolites in PMNCs. PCA showed a partial, unsupervised separation between controls and the OSAS group (PC1: 28.2%, PC2: 13.5%; total variance: 41.7%). The PLS-DA score plot is also provided for exploratory visualization (41% total variance); however, as demonstrated by permutation testing (see Supplementary Figure S4), this apparent forced separation lacks robust predictive significance (p = 0.50) and reflects the overfitting typical of high-dimensional, small-sample datasets.

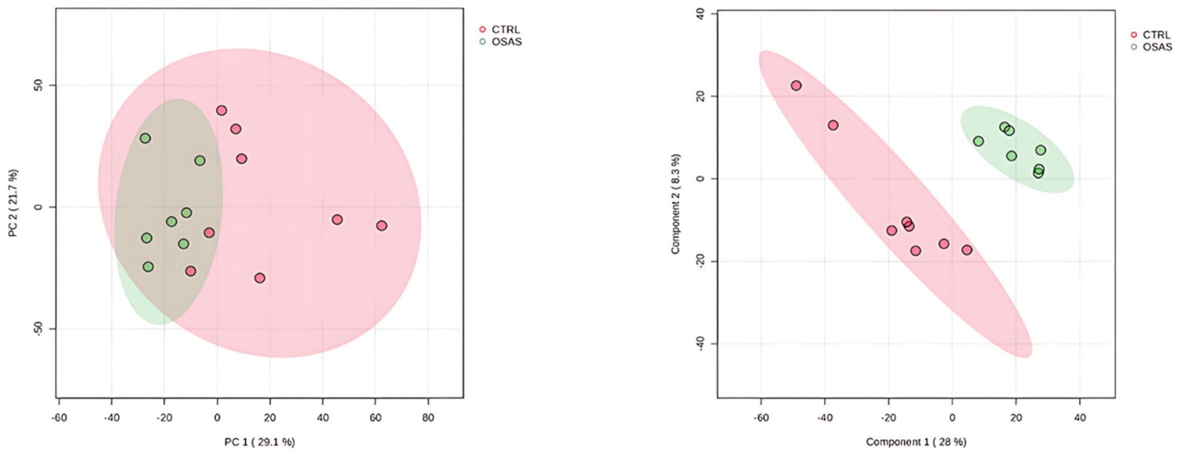

Supplementary Figure S5. Multivariate analysis of negative-ionization metabolites in PMNCs. PCA suggested a partial, unsupervised separation between the control group and the OSAS group, with PC1 and PC2 together accounting for 50.8% of the total variance. A PLS-DA score plot is also provided to visualize the maximal covariance between the metabolomic data and class labels (first two components: 36.3% variance); however, permutation testing (p = 0.50, see Supplementary Figure S4) confirmed that this supervised separation is not statistically robust and should be interpreted solely as an exploratory visual trend.

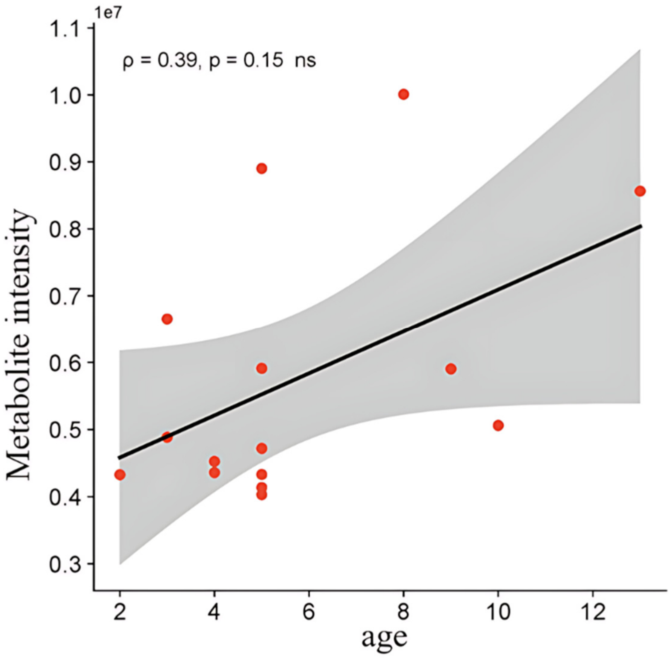

Supplementary Figure S6. Exploratory correlation analysis between age and cis,cis-muconic acid abundance. Spearman rank correlation analysis was evaluated across the full study cohort (n = 15, age range: 2–13 years). The analysis revealed a positive but statistically non-significant association between subject age and metabolite intensity (rho = 0.39, p = 0.15). The black solid line represents the linear regression trend, and the shaded gray area indicates the 95% confidence interval. While no statistically significant relationship with chronological age was detected, the limited sample size of this pilot study precludes excluding a modest contribution of age-related developmental stages to the observed variability in metabolite abundance.
