# Supplementary material for: Peripheral Mononuclear Cells Metabolomics in Obstructive Sleep Apnea Syndrome (OSAS): An Exploratory Pilot Study of Immunometabolic Signatures
Source: Int J Mol Sci. 2026 Aug 26;27(17):7658. doi: 10.3390/ijms27177658 (PMC13566245; doi:10.3390/ijms27177658)
Supplement: Supplementary file 1 [file ijms-27-07658-s001.zip › Supplementary file S4.pdf]

Supplementary File S4— Column Abbreviations and Definitions Table

- **Name:** Putative or confirmed chemical name assigned to the metabolic feature.
- **Formula:** Predicted molecular formula based on exact mass and isotopic pattern scoring.
- **Annot. DeltaMass [ppm]:** Mass accuracy error calculated as the difference between the observed mass-to-charge ratio (m/z) and the theoretical exact mass, expressed in parts per million (ppm).
- **Calc. MW:** Calculated neutral molecular weight of the compound (Da).
- **m/z:** Experimentally measured mass-to-charge ratio of the detected ion.
- **RT [min]:** Chromatographic retention time expressed in minutes.
- **Area (Max.):** Maximum peak area intensity integrated across the biological and quality control samples.
- **ChemSpider Hits:** Number of database matches returned by ChemSpider.
- **mzCloud Best Match Score:** mzCloud similarity score for the best MS/MS spectral match.
- **mzCloud Confidence:** Confidence level assigned to the mzCloud identification.
- **MS2:** Indicates whether MS/MS fragmentation evidence was available for the feature.
- **Reference Ion:** Reference ion used for annotation or alignment.
- **Fold Change:** Ratio of mean abundance between the OSAS and control groups.
- **Pvalue_MannWhitney:** Raw p-value from the Mann–Whitney U test.
- **q-value:** Benjamini–Hochberg false discovery rate (BH-FDR) adjusted q-value.
- **MSI Confidence Level:** Metabolomics Standards Initiative confidence level assigned to the annotation.
- **Sample Abundance Columns:** Normalized abundance values for each individual sample. Sample labels indicate group assignment (CTRL or OSAS).
